# Prenatal morphine exposure reduces pyramidal neurons in CA1, CA2 and CA3 subfields of mice hippocampus

**Published:** 2014-03

**Authors:** Soraya Ghafari, Mohammad Jafar Golalipour

**Affiliations:** 1 Department of Anatomical Sciences, Golestan University of Medical Sciences, Gorgan, Iran; 2 Gorgan Congenital Malformations Research Center, Department of Anatomical Sciences, Golestan University of Medical Sciences, Gorgan, Iran

**Keywords:** CA1, CA2, CA3, Hippocampus, Morphine sulfate, Mouse, Pyramidal cells

## Abstract

***Objective(s):*** This study was carried out to evaluate the effect of maternal morphine exposure during gestational and lactation period on pyramidal neurons of hippocampus in 18 and 32 day mice offspring.

***Materials and Methods:*** Thirty female mice were randomly allocated into cases and controls. In case group, animals received morphine sulfate 10 mg/kg.body weight intraperitoneally during 7 days before mating, gestational period (GD 0-21), 18 and 32 days after delivery in the experimental groups. The control animals received an equivalent volume of normal saline. Cerebrum of six offsprings in each group was removed and stained with cresyl violet and a monoclonal antibody NeuN for immunohistochemical detection of surviving pyramidal neurons. Quantitative computer-assisted morphometric study was done on hippocampus.

***Results: ***The number of pyramidal neurons in CA1, CA2 and CA3 in treated groups was significantly reduced in postnatal day 18 and 32 (P18, P32) compared to control groups (*P*<0.05).

The mean thickness of the stratum pyramidal layer was decreased in the treated groups in comparison with controls (*P*<0.05), whereas the mean thickness of the stratum oriens, stratum radiatum and stratum lacunosum-moleculare in CA1 field and stratum oriens, stratum lucidum, stratum radiatum and stratum lacunosum-moleculare in CA3 were significantly increased in morphine treated group in comparison with controls (*P*<0.05).

***Conclusion:*** Morphine administration before and during pregnancy and during lactation period causes pyramidal neurons loss in 18 and 32 days old infant mice.

## Introduction

Morphine (C_17_ H_19_ O_3_ N) is one of the 40 alkaloids which are present in opium from *Papaver somniferum* and is considered as one of the strongest known analgesic compounds (1). Worldwide, the prevalence of opioid abuse is high particularly in young people, morphine as one of the addictive drugs leads to increase cause of death, morbidity and lost productivity (2). Several studies have shown that opioid abuse may affect the embryos of pregnant women. In this regard, it has been shown that opioid administration during pregnancy caused delay in embryonic development, preterm labor, fetus death, chromo-somal anomalies, neural tube defects and reduced birth weight (3-8). Also, morphine neonatal abstinence is common in the infants of opioid dependent mothers (9). 

These children had several behavioral abnormalities including hyperactivity, lower Mental Development index, and lower motor development index (3, 4, 6). 

Several studies have reported that morphine has toxic effects on neurons in brain and spinal cord in animal model (10-14). 

The hippocampus is implicated in the control of several brain functions such as memory and learning and represents a neuronal structure with a high degree of functionality and cellular complexity.

Several studies have shown that opiates reduce hippocampal neurons (15), somatosensory neurons (16) and neurons in layer II/III in lateral secondary visual cortex (17) and inhibit neurogenesis in the adult rat hippocampus (18).

Furthermore, enhanced cell proliferation may be important for hippocampal-dependent learning and memory. Regarding to the high prevalence of opioid abuse in the world especially in young adult and little number of studies on the effect of morphine sulphate on neuronal development of hippocampus, the present study was carried out to clarify the neurotoxic effects of prenatal morphine sulphate administration on hippocampal neuronal density of mice neonates.

## Materials and Methods

This experimental study was performed at the Gorgan Faculty of Medicine, Golestan University of Medical Sciences, Gorgan, Iran. Guidelines on the care and use of laboratory animals and approval of the ethics Committee of Golestan University of Medical Sciences were obtained before the study.

***Experimental animals***

BALB/c mice, weighing 28–30 g (8–9 weeks old) were used in this study. The animals were maintained in a climate-controlled room under a 12 hr alternating light/dark cycle, 20°C to 22°C temperature. Dry food pellets and water were provided *ad libitum*.

***Drugs***

Each vial of l ml of morphine sulphate (Darou Pakhsh CO, Iran) was dissolved in 3.3 ml sterile saline solution (0.85%) to give 10 mg morphine sulphate to mice, intraperitoneally (IP). 

***Treatment groups***

After 2 weeks of acclimation to the diet and the environment, female mice were randomly divided into control and treated groups. Twelve female mice in treated group received 10 mg/kg body weight of morphine sulphate (IP) during 7 days before mating, gestational period (GD 0-21), 18 days after delivery in experimental group I and 32 days after delivery in experimental group II. 

Twelve female mice in control groups received an equivalent volume normal saline (IP) during 7 days before mating, gestational period (GD 0-21) 18 days after delivery in experimental group III and 32 days after delivery in experimental group IV.

After parturition, in each group, six postnatal days 18 and 32 (P18, P32) were randomly selected and killed quickly using chloroform anesthesia. The brain was exposed and fixed by immersion into the fixative solution (10% neutral-buffered formalin). After tissue processing, brains were sectioned at 6 micrometer thickness (an interval of 24 µm between every two consecutive sections) using a microtome (Microm HM 325, Germeny). The coronal sections (serial sections of anterior to posterior cerebrum) were serially selected -1.055 mm to -3.30 mm to bregma for hippocampal formation. Sections were used for immunohistochemistry and cresyl violet staining. 

***Immunohistochemistry***

Immunocytochemical labeling to detect the neuronal marker was performed by monoclonal anti-neuronal nuclei (NeuN) antibody (Millipore corporation Billerica, USA) on 5 μm thick hippocampal coronal sections.

In brief, deparaffinized sections were pre-incubated with citrate buffer and were washed for 9 min in 0.01 M phosphate-buffered saline (PBS, pH 7.4) and treated with 0.3% hydrogen peroxide in 0.01 M PBS including 10% methanol. The brain sections were preincubated with blocking reagent and washed in 0.01 M PBS. Then, brain sections were incubated with anti-NeuN antibody (1:100) in a humidified chamber for 1 hr at room temperature.

After rinse in 0.01 M PBS, the sections were incubated with the biotinylated secondary for 10 min and then with Streptavidin HRP and rinsed in PBS. Immunoreactivity was visualized using DAB (chromogen reagent) for 30 min at room temperature. Subsequently, the tissue specimen was counterstained with Mayer's hematoxylin and mounted with Entellan (Merck, USA).

**Figure 1:**
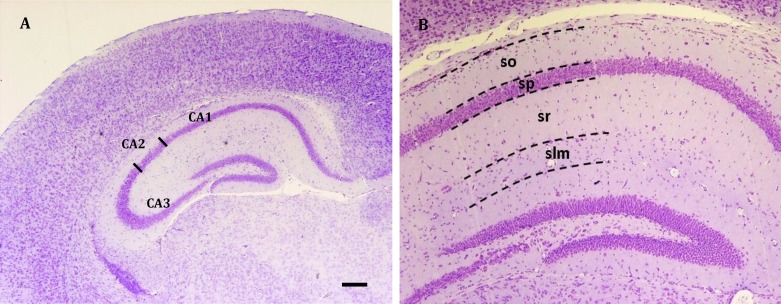
Overview of hippocampal areas used for quantitative measurements from BALB/c mice (P32) in control animal. Coronal sections stained with cresyl violet. A, Quantification areas are: CA1, cornu ammonis 1; CA2, cornu ammonis 2; CA3, cornu ammonis 3 medial part; (40X magnification, Scale bar: 200 μm). B, Layers of hippocampus; included stratum oriens (so), stratum pyramidal (sp), stratum radiatum (sr) and stratum lacunosum-moleculare (slm); (100X magnification)

**Figure 2:**
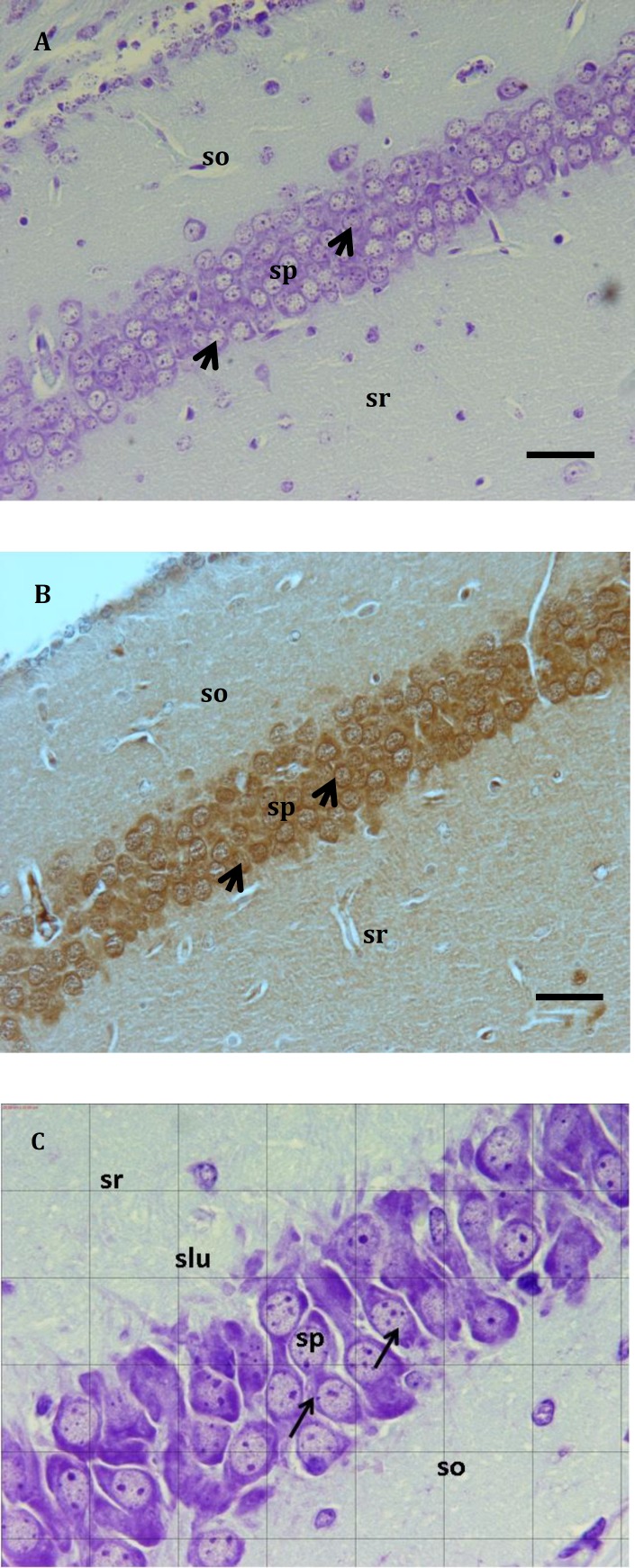
Hippocampal subfield in BALB/c mice (P32) control animal. A, Cresyl violet staining B, Neu-N positive neurons in CA1 region. (400X magnification, Scale bar: 40 μm). C, CA3 (1000X magnification, Grid: 20 μm ×20 μm). Arrows showing the pyramidal cells in (sp). stratum oriens (so), stratum pyramidal (sp), stratum radiatum (sr) and stratum lucidum (slu)

**Figure 3:**
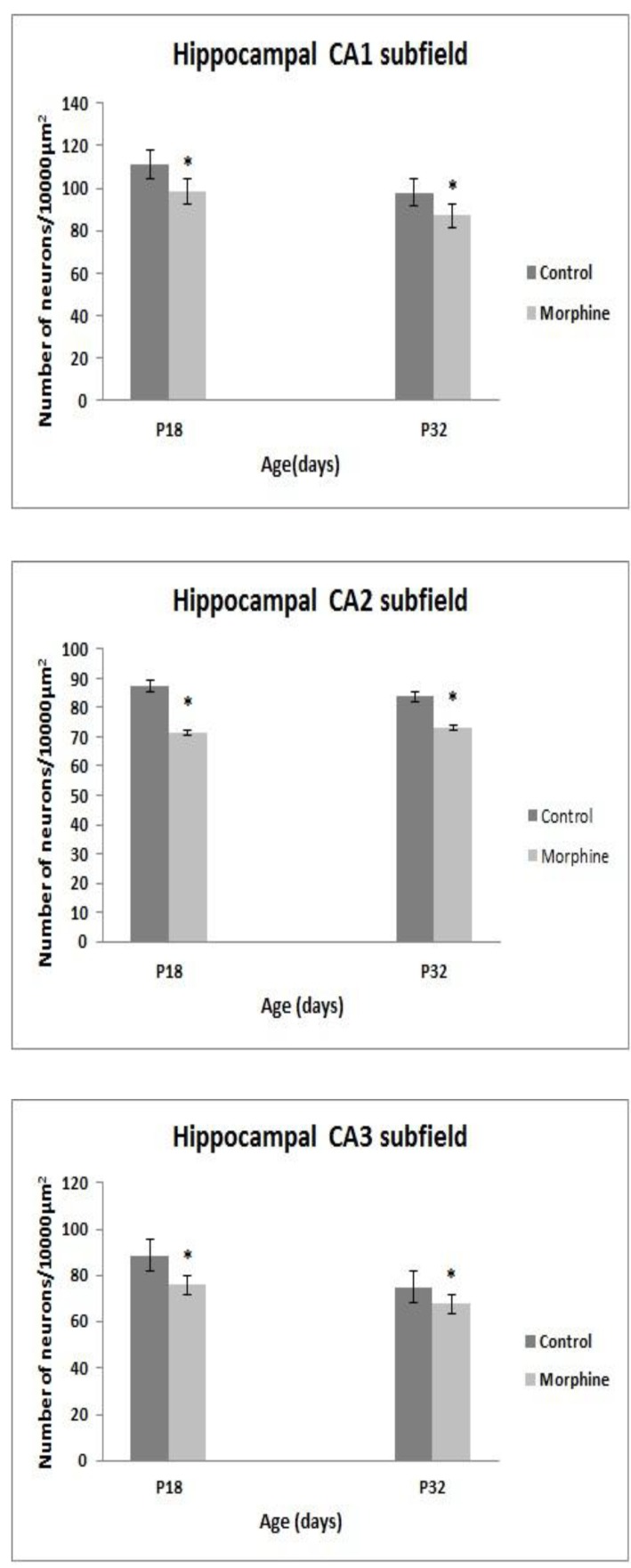
The mean number of pyramidal cells in (a) hippocampal CA1, (b) CA2 and (c) CA3 subfield in P18 and P32 mice of control and morphine sulphate treated mothers. The cells were expressed as the numb er of pyramidal cells per 10000 μm^2^, (results are means± SEM, *Compared with controls *P<*0.05, n=6)

**Table 1:** The thickness of the various layers of hippocampal subfield (μm) in postnatal day (P18) of morphine sulphate and control mothers

Hippocampal region		P18	
		Control	Morphine
CA1			
	Stratum oriens (so)	103.48±2.5	109.53±3.4
	Stratum pyramidal (sp)	56.42±2.3	49.41±0.3*
	Stratum radiatum (sr)	163.68±4.8	177.38±3.1*
	Stratum lacunosum-moleculare (slm)	62.70±2.9	79.18±3.3*
CA2			
	Stratum oriens (so)	92.88±4.2	109.18±3.9*
	Stratum pyramidal (sp)	57.93±4.3	52.86±3.2
	Stratum radiatum (sr)	151.05±5.6	159.31±4.8
	Stratum lacunosum-moleculare (slm)	57.36±1.6	60.5±1.8
CA3			
	Stratum oriens (so)	95.46±1.7	104.61±2.1*
	Stratum pyramidal (sp)	61.31±2.3	55.32±1.4*
	Stratum lucidum (slu)	35.56±1.2	41.98±1.6*
	Stratum radiatum (sr)	72.86±6.9	85.84±3.2
	Stratum lacunosum-moleculare (slm)	30.3±1.9	37.11±1.6*

Results are expressed as Mean±SEM of the mean

(*compared with control group, *P<*0.05, n=6)

***Morphometric analysis***

For histomorphometric study, the sections were observed under the light microscope. 

In each postnatal mouse, ten similar sections of anterior to posterior of CA1, CA2 and CA3 subfields of hippocampus were selected and images were taken and analyzed by Olympus BX 51 microscope and DP12 digital camera attached to OLYSIA autobioreport software (Olympus Optical, Co. LTD, Japan). The pyramidal cells density was evaluated by counting number of pyramidal cells per 10000 μm^2^ area of pyramidal layer of CA1, CA2 and CA3 subfield in 1000X magnification. The thickness of layers of hippocampus in CA1 and CA2 included stratum oriens (So), stratum pyramidal (Sp), stratum radiatum (Sr) and stratum lacunosum-moleculare (Slm) and in CA3 field included So, Sp, Sr, Slm and stratum lucidum (Slu) were obtained from 400X magnification (Figures 1-3).

***Statistical analysis***

Statistical analysis was done using the statistical package SPSS 16. All data are given as mean ± standard error of the mean (SEM). Comparisons between pairs of groups were carried out using Student's *t* test. Values of *P*<0.05 were considered to be statistically significant.

## Results

***The pyramidal neuron density***

In the P18 mice the numbers of NeuN positive neurons in CA1, CA2 and CA3 significantly reduced from 111.06±2.2, 87.34±3.0 and 88.63±3.0 cells in control groups to 98.55±3.1, 71.63±2.9 and 75.94±3.2 cells in 10000 μm^2^ area of pyramidal layer in treated groups (*P*<0.05) (Figures 1-3). 

In the P32 mice the numbers of NeuN positive pyramidal neurons in CA1, CA2 and CA3 significantly reduced from 97.87±4.1, 83.85±1.7 and 74.97±2.8 cells in control groups to 87.11±0.9, 73.30±3.0 and 67.71±1.4 cells in 10000 μm^2^ area of pyramidal layer in treated groups (*P*<0.05) (Figures 1-3).

**Table 2:** The thickness of the various layers of hippocampal subfield (μm) in postnatal day (P32) of morphine sulphate and control mothers

Hippocampal region		P32	
		Control	Morphine
CA1			
	Stratum oriens (so)	116.94±2.3	123.24±3.3
	Stratum pyramidal (sp)	60.52±1.9	51.90±2.1*
	Stratum radiatum (sr)	182.17±6.4	202.27±5*
	Stratum lacunosum-moleculare (slm)	87.74±3.3	99.26±3.6
CA2			
	Stratum oriens (so)	104.52±3.4	122.50±4.6
	Stratum pyramidal (sp)	54.42±3.3	43.46±2.3
	Stratum radiatum (sr)	192.68±3.8	199.14±5.1
	Stratum lacunosum-moleculare (slm)	50.42±2.1	58.3±1.6
CA3			
	Stratum oriens (so)	98.78±3.4	111.27±4.5
	Stratum pyramidal (sp)	64.19±2.4	50.41±2.9*
	Stratum lucidum (slu)	45.78±1.3	49.51±1.7
	Stratum radiatum (sr)	84.40±2.7	95.45±4.8
	Stratum lacunosum-moleculare (slm)	52.56±3.3	58.2±0.1.8

Results are expressed as Mean±SEM of the mean

(* compared with control group, *P<*0.05, n=6)

***The thickness of hippocampal CA1, CA2 and CA3 layers***

The thickness of the various layers of hippocampus (μm) in postnatal day (P18, P32) of morphine treated and controls is mentioned in Table 1, 2. The results revealed a significant reduction in the pyramidal layer thickness in CA1, CA2 and CA3 of treated mice compared to the controls in P18 and P32 mice (*P*<0.05), but the thickness of other layers increased in the treated group in comparison with control group in the postnatal 18 and 32 day mice (Table 1, 2). It means that the mean thickness of the stratum oriens (so), stratum radiatum (sr) and stratum lacunosum-moleculare (slm) in CA1 field and stratum oriens (so), stratum lucidum (slu), stratum radiatum (sr) and stratum lacunosum-moleculare (slm) in CA3 significantly increased in morphine treated group in comparison with controls (*P*<0.05). 

## Discussion

This study revealed that morphine sulfate administration before and during pregnancy and during lactation causes pyramidal cells loss, reduction of pyramidal layer thickness in 18 and 32 day old infant mice. Also, our study showed that even after lactation period, toxic effect of morphine continues on pyramidal cells of hippocampus.

Several studies have shown the toxic effects of morphine on neuronal cells in different parts of CNS either in adult or fetal period (10, 15-20). Prenatal morphine exposure impairs the juvenile offspring's dentate synaptic plasticity and spatial memory probably due to decreased GABAergic inhibition (19). Morphine induces apoptosis in neurons of rat's spinal cord (10). In an *in vitro* model, morphine reduced the number of hippocampal neurons that might be because of the induction of apoptosis in neurons of hippocampus (15). Morphine reduces the number of neurons in sematosensory of six day old rats (17) and the number of neurons in layer II/III in lateral secondary visual cortex of rats (16).

Moreover, long-term exposure to opiates inhibits neurogenesis in the adult rat hippocampus and chronic, but not acute, morphine reduces the number of BrdUrd-positive cells in the subgranular zone of the dentate gyrus (18). 

Furthermore, recent studies reported that morphine sulfate induces neurotoxicity on cerebellum and cerebral cortex (13, 14) and its oral administration (during gestational period), reduces both cortical thickness and the number of neurons in the developing fetal frontal cerebral cortex (13). Long-term exposure of dams to morphine sulfate significantly reduced the Purkinje cells number in mice offspring (14).

Indeed, in this study we observed the reduction of pyramidal layer thickness in all area of hippocampus which can be due to the decrease in pyramidal neurons in hippocampus. Regarding the effect of morphine on central nervous system, following possible mechanisms can be considered.

Loss of the neuronal cells in morphine treated animals can be due to apoptosis and or necrosis (21).

On the other hand, morphological alterations of astrocyte due to morphine can increase Ca^2+^ and induce production of carbonyl oxidation which subsequently promotes apoptosis and or necrosis in neurons (22).

Also, double-immunofluorescence staining method for the neuronal marker Neu-N and active caspase-3 and TUNEL assay showed that chronic morphine exposure induces apoptosis in neuronal cells (20).

Furthermore, Mao *et al* have shown that morphine increased Bax and Caspase -3 and reduced Bcl, indicators of apoptosis in neurons, in rats (10).

Also, the proliferation of neuroblasts in molecular layer can be blocked by morphine through DNA synthesis blocking (23).

Besides, several investigations reported that proliferation, differentiation and survival of neuroblasts and astroglia of cerebrum can be arrested by acute opioids exposure (24-28).

Also, the involvement of opioids in cerebral growth regulation has been revealed by experimentally perturbing the endogenous opioid system. Endogenous opioid peptides and receptors are widely expressed by developing cerebellar cells (29-33).

Neurotoxic effects of opioids can be induced by N-methyl-D-aspartate receptor (NMDAR)-caspase pathway. It has been suggested that N-methyl-D-aspartate (NMDA) receptors play a critical role in morphine-induced apoptosis in the superficial spinal cord dorsal horn of tolerant rats (10). 

Prolonged morphine administration also induces up-regulation of proapoptotic proteins caspase-3 and Bax as well as down-regulation of antiapoptotic protein Bcl-2. The general caspase inhibitor and caspase-3-specific inhibitor prevent morphine neurotoxicity (10). 

Also, Villarreal *et al* (2008) have shown that prenatal morphine exposure disrupts long-term potentiation (LTP) via disruption of opioid mechanisms involved in LTP maintenance or via disruption of opioid receptor activation during LTP induction, which can subsequently alter LTP maintenance (34).

Indeed, mitochondrial damage (35-37) and reduction of calbindin protein as a neuroprotective agent in neurons can be considered as possible mechanisms for the neuronal cells loss in hippocampus (38). Furthermore, it was suggested that opioids block the neuronal activity, causing the neurons to receive internal signals to commit suicide (apoptosis) (39).

## Conclusion

This study revealed that morphine administration before and during gestational and during lactation periods causes the neuronal cells loss and reduction of the layer of hippocampus in 18 and 32 day infant mice. Also, we can conclude that the neurotoxic effect of morphine will be continued even after exposure.
